# Berotralstat Tolerability and Effectiveness in Hereditary Angioedema: Berolife Study Results

**DOI:** 10.1002/clt2.70193

**Published:** 2026-08-15

**Authors:** Delphine Gobert, David Launay, Isabelle Boccon‐Gibod, Melisande Bourgoin‐Heck, Cyrille Hoarau, Yann Ollivier, Hervé Maillard, Fabien Pontille, Nicolas Ozanne, Magali Aubineau, Claire de Moreuil, Anne Gerber, Fabien Pelletier, Pierre‐Yves Jeandel, Aurélie Du‐Thanh, Marie‐Caroline Dalmas, Marie‐Caroline Taquet, Jean Schmidt, Amélie Servettaz, Amandine Perier, Enzo Cohen, Laure Carriat, Nicolas Lemaire, Mona Villedieu, Jean‐Charles Crave, Olivier Fain, Laurence Bouillet

**Affiliations:** ^1^ Sorbonne Université Internal Medicine Department AP‐HP Saint Antoine Hospital Paris France; ^2^ French National Reference Center for Angioedema (CRÉAK) Paris France; ^3^ Univ. Lille Inserm CHU Lille Lille France; ^4^ Institute for Translational Research in Inflammation (INFINITE) Lille France; ^5^ Department of Internal Medicine and Clinical Immunology CHU Lille French National Reference Center for Angioedema (CRÉAK) Lille France; ^6^ Internal Medicine Department Grenoble Alpes University Hospital French National Reference Center for Angioedema (CRÉAK) Grenoble France; ^7^ Department of Internal Medicine Saint‐Antoine Hospital French National Reference Center for Angioedema (CRÉAK) Paris France; ^8^ Allergy Department Trousseau Hospital Sorbonne Université Paris France; ^9^ Department of Allergology and Clinical Immunology Inserm UMR1327 ISCHEMIA Tours University Hospital University of Tours French National Reference Center for Angioedema (CRÉAK) Tours France; ^10^ Department of Allergy Caen University Hospital Caen France; ^11^ Department of Dermatology Le Mans Hospital Center Le Mans France; ^12^ Department of Internal Medicine Nancy University Hospital Nancy France; ^13^ Department of Internal Medicine Rouen University Hospital Rouen France; ^14^ Department of Internal Medicine Lyon University Hospital Lyon France; ^15^ Department of Internal Medicine Brest University Hospital Brest France; ^16^ Félix Guyon University Hospital La Reunion University Hospital Saint Denis La Réunion France; ^17^ Department of Dermatology and Allergology Besançon University Hospital Besançon France; ^18^ Department of Internal Medicine Nice University Hospital Nice France; ^19^ Department of Dermatology Montpellier University Hospital Montpellier France; ^20^ Department of Internal Medicine Hautepierre Hospital Strasbourg University Hospital Strasbourg France; ^21^ Department of Internal Medicine Strasbourg University Hospital French National Reference Center for Angioedema (CRÉAK) Strasbourg France; ^22^ Department of Internal Medicine Amiens‐Picardie University Hospital Amiens France; ^23^ Department of Internal Medicine and Clinical Immunology Reims University Hospital Reims France; ^24^ Department of Internal Medicine Niort University Hospital Niort France; ^25^ Medical Affairs Department BioCryst Pharmaceuticals France Boulogne‐Billancourt France; ^26^ Heva Lyon France; ^27^ BioCryst Pharmaceuticals, Inc. Durham North Carolina USA; ^28^ Internal Medicine Department Saint‐Antoine Hospital AP‐HP Sorbonne Université Paris France

**Keywords:** Berolife, berotralstat, hereditary angioedema, long‐term prophylaxis

## Abstract

**Background:**

Berotralstat is a first‐line once‐daily oral prophylactic treatment for hereditary angioedema (HAE). Berolife was designed to evaluate the tolerability and effectiveness of berotralstat in real‐world conditions.

**Methods:**

Berolife is an open‐label, multicenter, observational study conducted in France from September 2021 to January 2024. The primary objective was to assess tolerability, and secondary objectives included characterization of the treated population and assessment of berotralstat effectiveness. HAE attack rate was assessed before and after initiation of berotralstat using the paired Wilcoxon rank‐sum test.

**Results:**

Altogether, 80 patients were enrolled in the study (75 with HAE‐C1INH type 1 or 2 and 5 with HAE‐nC1INH) and received at least one dose of berotralstat. At baseline, patients had a mean (standard deviation [SD]) age of 40.0 (17.5) years, and most (67.5%) had received prior long‐term prophylaxis treatment. The mean (SD) duration of berotralstat treatment was 11.5 (8.5) months. Treatment‐related adverse events (AEs) occurred in 45.0% of patients, with gastrointestinal disorders being the most common (diarrhea: 13.8%, abdominal pain: 12.5%, upper abdominal pain: 7.5%). Importantly, no treatment‐related serious AEs were reported. A total of 61 patients were treated with berotralstat for ≥ 6 months and evaluated for effectiveness. At 6 months of treatment, a significant reduction in monthly HAE attack rates was observed (mean [SD]: 0.62 [0.66] vs. mean [SD]: 1.25 [1.10] at baseline; *p* = 0.001).

**Conclusions:**

Berotralstat was generally well tolerated with a tolerability profile consistent with prior clinical trials. Significant reductions in monthly HAE attack rates were observed, confirming its effectiveness in real‐world settings.

## 1 Introduction

Hereditary angioedema (HAE) is a rare genetic disorder characterized by spontaneous, recurrent, and potentially life‐threatening swelling attacks affecting the extremities, face, abdomen, and upper airways [1, 2]. HAE is commonly caused by a genetic variant in the *SERPING1* gene leading to a deficiency (HAE‐C1INH‐Type1) or dysfunction (HAE‐C1INH‐Type2) of the C1 inhibitor (C1‐INH) protein [1, 2]. This results in an accumulation of bradykinin leading to swelling attacks [3, 4]. Some patients are diagnosed with HAE with normal C1‐INH protein (HAE‐nC1INH) and experience HAE symptoms despite having normal levels and functioning of C1‐INH protein [3].

On‐demand, short‐term prophylaxis, and long‐term prophylaxis (LTP) treatments may be initiated to maintain control of HAE symptoms [5]. The French guidelines National Diagnostic and Care Protocols (PNDS) recommend that LTP treatment should be offered to any patient with HAE experiencing at least one attack per month, five severe attacks per year, or attacks that significant impact quality of life (QoL) [6]. The French National Plan on Rare Diseases 2018–2022 has played a pivotal role in advancing coordinated care and real‐world research for rare diseases such as HAE [7]. To prioritize equitable access to diagnosis and treatment, a nationwide network of expert centers dedicated to the diagnosis, management, and follow‐up of HAE patients was established, the National Reference Center for Angioedema (CRÉAK). Additionally, the plan has promoted the implementation of real‐world studies that reflect the lived experiences of patients.

In 2020, a French survey of HAE physicians from 25 different centers reported that 59.2% of patients with HAE were being treated with LTP; therapies included oral androgens (28.4%), progestins (25.8%), lanadelumab (25.3%), tranexamic acid (14.2%), and appropriate plasma‐derived C1‐INH formulations (6.2%) [8]. A patient survey indicated that half of patients with HAE described their prophylactic treatments as “burdensome” [9].

Berotralstat is a once‐daily oral inhibitor of plasma kallikrein approved for first‐line prophylaxis of HAE attacks in patients ≥ 12 years old [10]. In clinical trials, berotralstat has been shown to reduce monthly HAE attack rates and improve QoL in patients with HAE [11, 12, 13, 14]. Berotralstat is the only approved oral kallikrein inhibitor LTP for patients with HAE. Berotralstat was approved for use by the United States Food and Drug Administration (US FDA) in 2020 [10] and by the European Medicines Agency (EMA) in 2021 [15].

Berolife is the first and largest real‐world observational study in Europe investigating tolerability and effectiveness of berotralstat to date. Here, we report the results from patients with HAE aged ≥ 12 years treated with berotralstat in France.

## 2 Methods

### 2.1 Study Design

**FIGURE 1:**
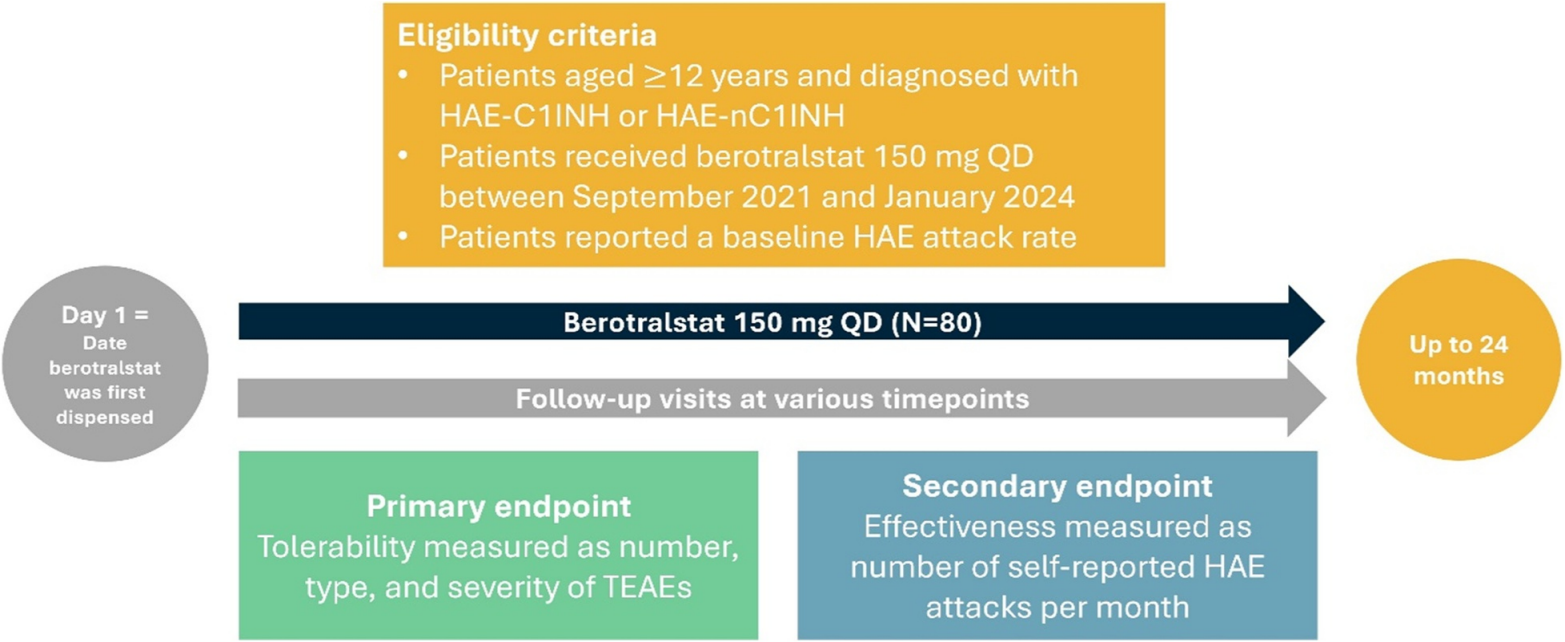
Berolife study design. HAE, hereditary angioedema; HAE‐C1INH, HAE with C1‐inhibitor deficiency; HAE‐nC1INH, HAE with normal C1‐INH protein; mg, milligrams; QD, once daily; TEAE, treatment‐emergent adverse event.

Berolife is a real‐world observational study for patients who received berotralstat treatment from September 2021 to January 2024 (Figure 1). A total of 22 CRÉAK centers were contacted and 20 accepted to participate in the study. Retrospective data were gathered from patient files, including confirmation of diagnosis, laboratory data, history of disease, and previous prophylactic treatment. Berotralstat treatment was prescribed as 150 mg once‐daily (QD) capsules. Day 1 was defined as the date that the prescription was issued by the physician.

Although not mandatory, follow‐up visits were suggested 1 month after treatment initiation and every 3 months thereafter. Data from follow‐up visits were categorized according to time ranges. Visits that occurred 0.5–1.5 months after treatment initiation were classified as Month 1. Thereafter, visits occurring within ± 1.5 months of discrete time ranges were classified according to those time points. For example, visits occurring 4.5–7.5 months after treatment initiation were classified as Month 6.

Adverse events (AEs) were assessed throughout the study for up to 24 months. AEs were independently assessed by the physician to determine severity and whether the AE was related to berotralstat treatment.

At treatment initiation and follow‐up visits, patients reported the number of HAE attacks. At baseline, a 30‐day baseline attack rate was determined by averaging the number of reported attacks during the 6‐month interval prior to enrollment. At follow‐up visits, a 30‐day attack rate was determined by averaging the number of HAE attacks since the last visit. Patients missing baseline HAE attack rate data were excluded from the effectiveness population but remained as part of the overall safety population.

### 2.2 Patients

Patients included in the Berolife study were aged 12 years and older, evaluated as outpatients, and seen in consultation by a participating physician. Enrolled patients had a confirmed diagnosis of HAE and met the approved indication for berotralstat for routine prevention of recurrent HAE attacks. Patients were treated with berotralstat 150 mg QD. Patients receiving at least one dose of berotralstat were included in the safety population; patients who remained on treatment for 6 months or more were included in the effectiveness population. The ≥ 6 month treatment criterion was selected to mimic a 24‐week endpoint in a clinical trial as well as to compare matching time periods (i.e., 6 months before treatment compared with 6 months after treatment). A small number of patients included in Berolife (*n* = 7) had previously received berotralstat through an early access program. All patients included in the study consented to the collection of personal health information and publication of anonymized data.

### 2.3 Objectives and Endpoints

The primary objective of the Berolife study was to describe the tolerability of berotralstat in a real‐world setting for up to 24 months. Secondary objectives included:

- Describe and characterize the study population.
- Assess the effectiveness of berotralstat using frequency of HAE attacks.

### 2.4 Statistical Analyses

For tolerability, reports of AEs will be provided using *n*, missing *n*, frequency, and percentage. Descriptive statistics will be provided throughout. For effectiveness outcomes, HAE attack rates will be provided using *n*, missing *n*, mean, standard deviation (SD), median, first and third quartiles (Q1 and Q3), and minimum and maximum (min, max). Type 1 error, *α*, was set at 5% for all *t*‐tests. A paired Wilcoxon test was used to assess the differences in attack rate prior to starting berotralstat and at 6 months follow‐up. Statistical analyses were performed using SAS software (SAS Institute, NC, USA) version 9.4.

## 3 Results

### 3.1 Study Populations

**FIGURE 2:**
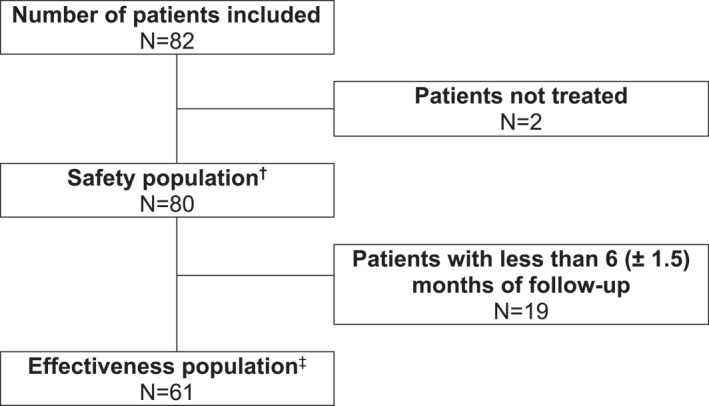
CONSORT diagram. ^†^The safety population refers to all patients included and treated with berotralstat. ^‡^The effectiveness population refers to all patients in the safety population with at least 6 months (± 1.5 months) of follow‐up.

As of the final data cut on August 23, 2024, 82 patients were enrolled in Berolife (Figure 2). Two patients were prescribed berotralstat but not treated and were thus excluded. Therefore, 80 patients were included in the safety population. Most patients in the safety population were female (57.5%) with a mean (SD) age of 40.0 (17.5) years (Table 1). Most patients were diagnosed with HAE‐C1INH‐Type1 (88.8%), 5.0% were diagnosed with HAE‐C1INH‐Type2, and 6.3% with HAE‐nC1INH with factor XII or plasminogen mutations. Patients had a mean (SD) age at diagnosis of 21.2 (15.1) years, and most patients reported a family history of HAE (82.5%). The most common comorbidities included hypertension (12.5%) and diabetes (6.3%). Most patients (67.5%) had previously used LTP at some point in their lifetime, and the most common previous LTPs were danazol (37.5%), tranexamic acid (27.5%), lanadelumab (10.0%), and progestins (3.8%).

**TABLE 1:** Baseline characteristics.

	Safety population (*N* = 80)	Effectiveness population (*n* = 61)
Gender, *n* (%)		
Female	46 (57.5)	31 (50.8)
Male	34 (42.5)	30 (49.2)
Age, years		
Mean (SD)	40.0 (17.5)	37.1 (17.2)
Median (min, max)	41 (11, 77)	38 (11, 76)
Comorbidities, *n* (%)		
No	62 (78.5)^a^	50 (83.3)^e^
Yes	17 (21.5)^a^	10 (16.7)^e^
Hypertension	10 (58.8)^b^	5 (50.0)^f^
Renal failure	1 (5.9)^b^	0^f^
Hepatic insufficiency	0^b^	0^f^
Heart failure	0^b^	0^f^
Diabetes	5 (29.4)^b^	3 (30.0)^f^
Concomitant treatment, *n* (%)		
No	49 (62.0)^a^	39 (65.0)^e^
Yes	30 (38.0)^a^	21 (35.0)^e^
CYP2D5 or P‐glycoprotein substrates	2 (6.7)^c^	2 (9.5)^g^
Diagnosis, *n* (%)		
HAE‐C1INH‐Type1	71 (88.8)	54 (88.5)
HAE‐C1INH‐Type2	4 (5.0)	3 (4.9)
HAE‐nC1INH	5 (6.3)	4 (6.6)
Family history of HAE, *n* (%)	66 (82.5)	50 (82.0)
HAE attack rate before initiation visit (at baseline), attacks/month		
Mean (SD)	1.1 (1.0)^d^	1.2 (1.1)^h^
Median (min, max)	0.8 (0.0, 4.0)^d^	0.9 (0.0, 4.0)^h^
Number of HAE attacks over 6 months at baseline, *n* (%)		
0–1 attacks	14 (17.5)	11 (18.0)
≥ 2 attacks	66 (82.5)	50 (82.0)
Previous preventative treatments	54 (67.5)	40 (65.6)
Danazol	30 (37.5)	22 (36.1)
Tranexamic acid	22 (27.5)	16 (26.2)
Lanadelumab	8 (10.0)	7 (11.5)
Complement C1 esterase inhibitor	1 (1.3)	0
Progestin derivatives	3 (3.8)	2 (3.3)

Abbreviations: CYP2D5, cytochrome P450 family 2 subfamily D polypeptide 5; FXII, factor XII; HAE, hereditary angioedema; HAE‐C1INH‐Type1, HAE with deficiency of the C1‐INH protein; HAE‐C1INH‐Type2, HAE with dysfunction of the C1‐INH protein; HAE‐nC1INH, HAE with normal C1‐inhibitor protein; max, maximum; min, minimum; PLG, plasminogen; SD, standard deviation.

^a^ Based on *n* = 79. One patient had missing data.

^b^ Based on *n* = 17 patients who had comorbidities.

^c^ Based on *n* = 30 patients who received concomitant treatment.

^d^ Based on *n* = 78 patients with available attack data.

^e^ Based on *n* = 60. One patient had missing data.

^f^ Based on *n* = 10 patients who had comorbidities.

^g^ Based on *n* = 21 patients who received concomitant treatment.

^h^ Based on *n* = 60 patients with available attack data.

The mean (SD) duration of berotralstat treatment was 11.5 (8.5) months, ranging from 0 to 27 months. Duration was defined as the treatment initiation date subtracted from the last visit date. Duration was 0 months for two patients (2.5%) because no follow‐up visits were performed. A total of 61 patients completed at least 6 months of follow‐up after berotralstat initiation and were included in the effectiveness population (Table 1). For this patient population, approximately half of the patients were female (50.8%) with a mean (SD) age of 37.1 (17.2) years. Most patients were diagnosed with HAE‐C1INH‐Type1 (88.5%) with a mean (SD) age at diagnosis of 19.8 (15.9) years; 82.0% of patients reported a family history of HAE. Similar to the safety population, the most common comorbidities in the effectiveness population included hypertension (8.2%) and diabetes (4.9%). Most patients had previously used LTP during their lifetime (65.6%), and the most common previous LTPs were danazol (36.1%), tranexamic acid (26.2%), lanadelumab (11.5%), and progestins (3.3%).

### 3.2 Tolerability

Half of all patients (40/80; 50.0%) reported at least one treatment‐emergent adverse event (TEAE; Table 2). Two patients reported serious TEAEs (2.5%; sputum purulent and paresthesia). No patients reported a treatment‐related serious AE. A total of 36/80 (45.0%) patients experienced a TEAE that was determined to be treatment‐related. Gastrointestinal (GI) disorders (Table 3) were among the most commonly reported treatment‐related AEs, which included diarrhea (13.8%), abdominal pain (12.5%), and upper abdominal pain (7.5%). GI disorders were reported by 21 patients during the first month after berotralstat treatment initiation. Between the first month and < 12 months of treatment, 12 patients reported GI disorders, showing that the percentage of patients with GI AEs significantly decreased after the first month of berotralstat treatment.

**TABLE 2:** Summary of adverse events.

	Analysis population
	Total (*N* = 80)
All TEAEs, *n* (%)	40 (50.0)
Serious TEAEs	2 (2.5)
All TEAEs related to berotralstat treatment, *n* (%)	36 (45.0)
TEAEs related to berotralstat treatment reported in > 5% of patients, *n* (%)	
Diarrhea	11 (13.8)
Abdominal pain	10 (12.5)
Abdominal pain upper	6 (7.5)
Serious TEAEs related to berotralstat treatment, *n* (%)	0 (0.0)
All treatment discontinuations due to TEAEs, *n* (%)	7 (8.8)

Abbreviation: TEAE, treatment‐emergent adverse event.

**TABLE 3:** TEAEs related (related, probably, possibly, suspect) to berotralstat treatment by MedDRA terms.

MedDRA SOC/PT term	Total (*N* = 80)		
	Nb of AEs	Nb of patients with at least one AE	Percentage of patients with at least one AE
Total	59	36	45.0
Gastrointestinal disorders	47	32	40.0
Diarrhea	11	11	13.8
Abdominal pain	10	10	12.5
Abdominal pain upper	6	6	7.5
Nausea	4	4	5.0
Abdominal distension	3	3	3.8
Abdominal discomfort	2	2	2.5
Gastrointestinal disorder	2	2	2.5
Abdominal rigidity	1	1	1.3
Constipation	1	1	1.3
Dyskinesia esophageal	1	1	1.3
Dyspepsia	1	1	1.3
Gastrointestinal motility disorder	1	1	1.3
Gastroesophageal reflux disease	2	1	1.3
Vomiting	1	1	1.3
Oral discomfort	1	1	1.3
Skin and subcutaneous tissue disorders	4	4	5.0
Rash	2	2	2.5
Erythema	1	1	1.3
Rash macular	1	1	1.3
General disorders and administration site conditions	3	3	3.8
Drug intolerance	3	3	3.8
Psychiatric disorders	2	2	2.5
Insomnia	1	1	1.3
Nervousness	1	1	1.3
Nervous system disorders	1	1	1.3
Headache	1	1	1.3
Reproductive system and breast disorders	2	1	1.3
Dysmenorrhea	2	1	1.3
Gastrointestinal disorder adverse events			
During the first month after treatment initiation	28	21	26.3
Between 1 and 3 months after treatment initiation	7	6	7.5
Between 3 and 6 months after treatment initiation	3	3	3.8
Between 6 and 9 months after treatment initiation	2	1	1.3
Between 9 and 12 months after treatment initiation	2	2	2.5
12 months or more after treatment initiation	0	0	0

Abbreviations: AE, adverse event; MedDRA, Medical Dictionary for Regulatory Activities; PT, preferred terms; SOC, system organ class; TEAE, treatment‐emergent adverse event.

During the first year of follow‐up, 30/80 (37.5%) patients discontinued berotralstat treatment due to AEs (7/30: 23.3%), insufficient perceived efficacy (13/30: 43.3%), poor adherence (9/30: 30%), or pregnancy (1/30: 3.3%). Among the 7 patients who stopped treatment because of TEAEs, 3, 3, and 1 discontinued between 0.5 and 1.5 months, between 1.5 and 4.5 months, and between 4.5 and 7.5 months, respectively. In the second year, 5 patients (6.3%) discontinued treatment, although these were not because of tolerability issues. Additionally, 2 more patients discontinued at an unknown time point.

### 3.3 Effectiveness

**FIGURE 3:**
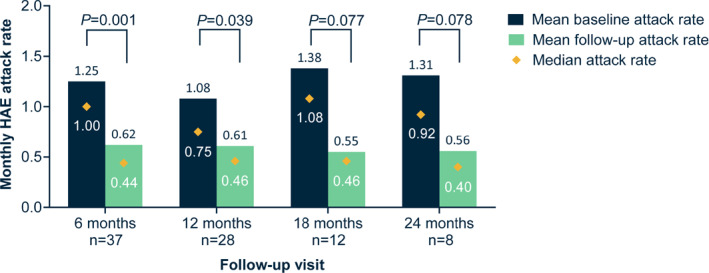
Monthly HAE attack rate at follow‐up visits compared with baseline. As follow‐up visits were not required, only the number of patients that participated in the follow‐up visit was included. HAE, hereditary angioedema.

Patients included in the effectiveness analysis (*n* = 61) reported their monthly HAE attacks (Figure 3). Patients reported a mean (median; *n*) of 1.25 (1.00; 37) HAE attacks per month at baseline, which was reduced to a mean (median; *n*) of 0.62 (0.44; 37; *p* = 0.001) HAE attacks per month at Month 6. Similarly, a significant reduction at Month 12 (mean 0.61; median 0.46; *n* = 28) was observed compared to baseline (mean 1.08; median 0.75; *n* = 28; *p* = 0.039).

## 4 Discussion

To date, the Berolife study is the first and largest observational study in Europe investigating the tolerability and effectiveness of berotralstat in a real‐world clinical setting. Patient‐reported TEAEs were similar to those reported in open‐label clinical trials, with diarrhea, abdominal pain, and upper abdominal pain among the most commonly reported.

In the effectiveness population, a statistically significant reduction in monthly HAE attack rates was observed at Months 6 and 12 of berotralstat treatment compared with baseline.

### 4.1 Comparison Between Berolife and Clinical Trial Data

Patients enrolled in Berolife exhibited similar mean age and gender distributions compared to the APeX‐2 and APeX‐S clinical trials [12, 13, 14]. The frequency of TEAEs between Berolife and both clinical trials were similar, with the majority of common TEAEs (abdominal pain and diarrhea) occurring in the earlier stages of treatment. However, mean baseline monthly HAE attack rates differed between studies; the mean baseline monthly HAE attack rate in Berolife was 1.1 (*N* = 80) compared with 3.1 in APeX‐2 (*N* = 81) [13]. Importantly, patients in Berolife were not required to wash out previous HAE treatments prior to enrollment, whereas patients in APeX‐2 were not using LTP prior to enrollment. In APeX‐2, of the 70 patients who reached 24 months of treatment; mean monthly attack rates at Month 6 ranged from 0.5 to 1.1, depending on the cohort [13]. In Berolife, the mean monthly attack rate at Month 6 was 0.62 (*n* = 37), similar to those reported in APeX‐2, and a statistically significant reduction from baseline. Despite study differences, Berolife aligned with APeX‐2 regarding tolerability and reduction in HAE attack rates [12, 13].

### 4.2 Comparison Between Berolife and Other Real‐World Evidence Studies

The Berolife study results also align with other real‐world evidence studies involving berotralstat. One small study using patient surveys reported that of the 10 patients on berotralstat, only 2 reported an unsatisfactory response and discontinued therapy [16]. Similarly, studies utilizing pharmacy data showed that patients treated with berotralstat had a significant and sustained reduction in HAE attack rates compared with baseline [17, 18, 19]. In a real‐world outcomes study, patients had similar baseline attack rates compared to patients enrolled in Berolife [20]. However, even among patients with low baseline attack rates, the median monthly attack rate following 90 days of berotralstat treatment was significantly decreased. These results, in addition to those from Berolife, demonstrate that berotralstat treatment provides a sustained reduction in HAE attack rates, regardless of baseline attacks. Lastly, results from the early access to medicines scheme protocol implemented in the UK in 2020 align with data from Berolife in that very few mild AEs and a low incidence of discontinuations due to AEs were reported [21].

### 4.3 Comorbidities in HAE

In the general French population, one third of adults have hypertension [22]. In Berolife, the incidence is lower, with 12.5% of patients having hypertension. Registry data from Sweden show that patients with HAE experience increased risk of cardiovascular disease, hypertension, hyperlipidemia, and autoimmune disease [23]. These comorbidities are likely associated with the use of androgens as LTP, as androgens are known to increase atherosclerosis and cardiovascular risk. Therefore, it is important for patients to have treatment options besides androgens.

### 4.4 Adherence

Although adherence was not assessed as part of the Berolife protocol, another French study, MATCH, has demonstrated durable adherence to berotralstat over extended treatment periods [24]. Furthermore, patient persistence rates with berotralstat have been shown to be comparable to other LTP options [19]. Real‐world experience indicates that initial training, education, and support may be required as part of the shared decision‐making process when implementing a new LTP.

### 4.5 Limitations

Some limitations of the Berolife study should be considered when reviewing the data. First, 26% of patients were enrolled from a single center. This “center effect” may introduce bias into the data. The inherent limitations of retrospective and observational study designs, which include lack of controlled variables, inconsistent patient reporting, and recall bias, may be present in Berolife. Additionally, the lack of mandatory visits in Berolife may lead to inconsistencies in the data and difficulties in drawing conclusions over time. The baseline attack rate was determined by averaging the number of reported attacks during the previous 6‐month interval. Since HAE is a very heterogenous disease and disease activity can vary, this short duration may not be representative of the overall attack rate. Moreover, a 3‐month window was allowed for each visit (e.g., visits occurring from 4.5 to 7.5 months after treatment initiation were classified as Month 6), which reduces the reliability of the number of attacks in each interval. This makes the interpretation of results and the comparison with phase‐3 clinical trials less relevant. Lastly, it is unclear whether the low baseline attack rates reported here are due to patients switching from a previous LTP to berotralstat treatment with the overall goal of maintaining efficacy while improving convenience or tolerability. Since no wash‐out period was required, the low baseline attack rates could have resulted from patients being on a prior LTP. However, detailed data regarding patients switching from a previous LTP to berotralstat (along with the reasons for switching) or being treatment‐free at berotralstat initiation were not collected. While this may be a missed opportunity, further investigations could gather and analyze such data.

## 5 Conclusion

The Berolife study highlighted that LTP with oral berotralstat was generally well tolerated and reports of AEs related to tolerability were consistent with those observed in clinical trials. Berotralstat therapy also led to a reduction in monthly HAE attack rates. These results confirm the tolerability and effectiveness of berotralstat in a real‐world setting, making it a verified option as an oral LTP for patients with HAE.

## Author Contributions

**Delphine Gobert:** investigation, writing – review and editing. **David Launay:** investigation, writing – review and editing. **Isabelle Boccon‐Gibod:** investigation, writing – review and editing. **Melisande Bourgoin‐Heck:** investigation, writing – review and editing. **Cyrille Hoarau:** investigation, writing – review and editing. **Yann Ollivier:** investigation, writing – review and editing. **Hervé Maillard:** investigation, writing – review and editing. **Fabien Pontille:** investigation, writing – review and editing. **Nicolas Ozanne:** investigation, writing – review and editing. **Magali Aubineau:** investigation, writing – review and editing. **Claire de Moreuil:** investigation, writing – review and editing. **Anne Gerber:** investigation, writing – review and editing. **Fabien Pelletier:** investigation, writing – review and editing. **Pierre‐Yves Jeandel:** investigation, writing – review and editing. **Aurélie Du‐Thanh:** investigation, writing – review and editing. **Marie‐Caroline Dalmas:** investigation, writing – review and editing. **Marie‐Caroline Taquet:** investigation, writing – review and editing. **Jean Schmidt:** investigation, writing – review and editing. **Amélie Servettaz:** investigation, writing – review and editing. **Amandine Perier:** investigation, writing – review and editing. **Enzo Cohen:** conceptualization, writing – original draft, writing – review and editing. **Laure Carriat:** investigation, writing – review and editing. **Nicolas Lemaire:** investigation, writing – review and editing. **Mona Villedieu:** conceptualization, writing – original draft, writing – review and editing. **Jean‐Charles Crave:** conceptualization, writing – original draft, writing – review and editing. **Olivier Fain:** investigation, writing – review and editing. **Laurence Bouillet:** investigation, writing – review and editing.

## Funding

This study was sponsored by BioCryst Pharmaceuticals Inc., Durham, NC, USA. The authors declare that funding from BioCryst Pharmaceuticals Inc., Durham, NC, USA, was also provided for medical writing support, editorial assistance, and article processing charges. The sponsor was involved in designing the study and in analyzing and interpreting the data with the authors.

## Ethics Statement

According to the research protocol, a designated Scientific Coordinator, Prof Laurence Bouillet, was responsible for validating the relevance of this study: objectives, methodology, and scientific quality of the project. He provided support in the study design, contributed to the drafting of the protocol, organized meetings as needed, and contributed to the study's publications. No ethics approval from an institutional review board was sought. Patients provided written consent prior to their participation.

## Consent

Patients provided consent to publish their de‐identified personal and medical information in a medical journal.

## Conflicts of Interest

D.G.: Advisory boards or speaker bureaus for BioCryst Pharmaceuticals Inc., CSL Behring, Pharvaris, and Takeda. D.L.: Received fees from BioCryst Pharmaceuticals Inc., CSL Behring, and Takeda. I.B.‐G.: Research funding from and advisor for BioCryst Pharmaceuticals Inc., CSL Behring, Ionis, KalVista, Otsuka, Pharming, and Takeda. M.B.‐H.: On advisory boards or speaker bureaus for ALK, BioCryst Pharmaceuticals Inc., CSL Behring, and Takeda. C.H.: Investigator in clinical trials for ALK, BioCryst Pharmaceuticals Inc., CSL Behring, LFB, and Stallergenes Greer; consultant/advisor for ALK, BioCryst Pharmaceuticals Inc., Blueprint Medicines, Octapharma, Pilège, Sanofi, Stallergenes Greer, and Takeda; research support from ALK and Stallergenes Greer. Y.O.: Financial support from BioCryst Pharmaceuticals Inc.; Takeda for congress participation. H.M.: Nothing to declare. F.Po.: Financial support from BioCryst Pharmaceuticals Inc.; CSL Behring, Takeda for congress participation. N.O.: Nothing to declare. M.A.: Receives fees from BioCryst Pharmaceuticals Inc. and Takeda. C.d.M.: Nothing to declare. A.G.: Nothing to declare. F.Pe.: Contracts, consultations, and/or support for attending meetings with BioCryst Pharmaceuticals Inc., CSL Behring, Otsuka, and Takeda. P.‐Y.J.: Speaker bureaus for BioCryst Pharmaceuticals Inc. and Takeda. A.D.‐T.: Consulted/served as speaker for, engaged in research projects with, or accepted travel grants from the following companies: BioCryst Pharmaceuticals Inc., Takeda, Novartis, GSK, KalVista, Pharvaris, Celltrion, ADARx Pharmaceuticals. M.‐C.D.: Receives fees from BioCryst Pharmaceuticals Inc. and Takeda. M.‐C.T.: Receives fees from Takeda. J.S.: Received fees from GSK and AstraZeneca. A.S.: Nothing to declare. A.P.: Nothing to declare. E.C.: Former employee of BioCryst Pharmaceuticals Inc.; Current employee of Neopharmed Gentili. L.C.: Former employee of BioCryst Pharmaceuticals Inc.; Current employee of Neopharmed Gentili. N.L.: Statistical consultant of Heva. M.V.: Former employee of BioCryst Pharmaceuticals Inc.; Current employee of Neopharmed Gentili. J.‐C.C.: Former employee of BioCryst Pharmaceuticals Inc.; Current employee of Neopharmed Gentili. O.F.: Receives consulting fees and honoraria from BioCryst Pharmaceuticals Inc., CSL Behring, Otsuka, and/or Takeda. L.B.: Consulted/served as speaker for, engaged in research and educational projects with, or accepted travel grants from the following companies: BioCryst Pharmaceuticals Inc., CSL Behring, Takeda, Blueprint Medicines, KalVista, Pharvaris, and Otsuka.

## Data Availability

The data that support the findings of this study are available from the corresponding author upon reasonable request.
